# A time series model of the occurrence of gastric dilatation-volvulus in a population of dogs

**DOI:** 10.1186/1746-6148-5-12

**Published:** 2009-04-15

**Authors:** Michael Levine, George E Moore

**Affiliations:** 1 Department of Statistics, College of Science, Purdue University, 250 N. University Street, West Lafayette, IN 47907, USA; 2 Department of Comparative Pathobiology, School of Veterinary Medicine, Purdue University, 725 Harrison Street, West Lafayette, IN 47907, USA

## Abstract

**Background:**

Gastric dilatation-volvulus (GDV) is a life-threatening condition of mammals, with increased risk in large breed dogs. The study of its etiological factors is difficult due to the variety of possible living conditions. The association between meteorological events and the occurrence of GDV has been postulated but remains unclear. This study introduces the binary time series approach to the investigation of the possible meteorological risk factors for GDV. The data collected in a population of high-risk working dogs in Texas was used.

**Results:**

Minimum and maximum daily atmospheric pressure on the day of GDV event and the maximum daily atmospheric pressure on the day before the GDV event were positively associated with the probability of GDV. All of the odds/multiplicative factors of a day being GDV day were interpreted conditionally on the past GDV occurrences. There was minimal difference between the binary and Poisson general linear models.

**Conclusion:**

Time series modeling provided a novel method for evaluating the association between meteorological variables and GDV in a large population of dogs. Appropriate application of this method was enhanced by a common environment for the dogs and availability of meteorological data. The potential interaction between weather changes and patient risk factors for GDV deserves further investigation.

## Background

Gastric dilatation-volvulus (GDV) is a condition in which the stomach dilates and rotates on itself, leading progressively to hypotension, shock, and death. Large breed dogs are frequently affected, although it can affect many animal species including humans [1].

The physical mechanisms involved in this condition and its treatment are well understood, but the causes are not [2]. Causes of GDV may be considered predisposing (increasing the likelihood of disease) or precipitating (triggering the onset of disease). Several predisposing risk factors in dogs have been suggested, including temperament of the dog (excitability), large or giant breed, increased thoracic depth to width ratio, and rapid food consumption [2-4]. However, many questions related to the actual onset of this life-threatening disease remain unexplained by these risk factors.

The study of precipitating causes for GDV is an important and not very well researched area. In many cases of GDV, the stomach is distended with gas; among possible sources of this gas, aerophagia, fermentation-putrefaction, chemical gas genesis and gas diffusion have been suggested. This, coupled with observed seasonal variation in GDV cases, gave rise to suspicions about the possible association between GDV and weather conditions shortly before its occurrence [5,6]. Methods applied by Herbold et al in [5], i.e. principal components analysis, to select possibly important climatologic factors may have obscured the impact of a single weather-related variable. Other studies have used logistic regression to investigate the probability of a day being a GDV day, given certain atmospheric pressure or temperature conditions [6,7]. Such approaches are based on a premise that GDV events are mutually independent and there is no strong correlation between events over time, as would occur if the disease were infectious. However, as a matter of fact, most meteorological covariates thought to influence GDV occurrence, are autocorrelated over time and should be thought of as time series. If some of them have not been included in a potential model, but they do influence the GDV occurrence, GDV incidences recorded over time may be correlated.

Thus, viewing the GDV occurrence data as time series may be advisable in practice. Although traditional time series modeling has had limited applicability in the study of uncommon diseases, it has been recently used to demonstrate a seasonal component to another gastrointestinal disease, namely, colic in horses [8]. Since the GDV occurrence data is clearly integer valued (the value of the response is the number of GDV cases per day), the more traditional time series methods, such as those used in the classical Autoregressive Integrated Moving Average (ARIMA) framework [9] cannot be used. One of the earlier references [8] suggests that"...One possibility lies in the use of a Poisson distribution to model count data within a framework broadly analogous to that of generalized linear modeling..."; this suggestion effectively amounts to using the approach based on generalized linear model (GLM) framework that is utilized in this manuscript.

The objective of this study was to use a time-series approach to investigate the association between meteorological variables and GDV occurrence in dogs. To reduce the variability of predisposing risk factors and confounding variables such as diet and housing environment [2], the dataset of large breed dogs housed at the Military Working Dog (MWD) Training Center at Lackland Air Force Base (LAFB) was used. In this common environment, the dogs are fed a standard diet, housed in outdoor runs, and are under observation 24 hrs a day. This dataset has been used before in a logistic regression approach to investigating meteorological variables and GDV [7], and a comparison of the methodological approaches can therefore also be made.

## Results

Several models that had some of the lowest values of AIC (Akaike Information Criterion) are shown in the Table 1. For each of them, the systematic part is given, together with the type of the model (binary GLM or Poisson GLM) and values of AIC. Only models that had log-likelihood ratio p-values of all covariates below 0.10 had been included. Since the dog census varies from day to day, the amount of exposure present is variable. This may make strictly Poisson/binary assumption about the data unrealistic. In the Poisson case, this means that the variance may not be exactly equal to the mean. In the binary case, the variance may not be exactly equal to the variance of Bernoulli distribution for a given set of covariate values. Thus, it seems reasonable to check for possible overdispersion in the data. A simple quasi-likelihood approach was used to look for evidence of overdispersion [10]. The estimated coefficient is always close to 1 and thus there does not seem to be any serious evidence of overdispersion in this setting.

**Table 1:** The Final Models

Model	Systematic part	Type of the model	AIC	Overdispersion factor
1	*β*_0 _+ *β*_1_*Y*_*t*-2 _+ *β*_2_*pmin*_*t*_	Binary GLM	411.02	1.02

2	*β*_0 _+ *β*_1_*Y*_*t*-2 _+ *β*_2_*pmin*_*t*_	Poisson GLM	407.92	0.98

3	*β*_0 _+ *β*_1_*Y*_*t*-2 _+ *β*_2_*pmax*_*t*-1_	Binary GLM	406.47	1

4	*β*_0 _+ *β*_1_*Y*_*t*-2 _+ *β*_2_*pmax*_*t*-1_	Poisson GLM	403.42	0.95

5	*β*_0 _+ *β*_1_*Y*_*t*-2 _+ *β*_2_*pmax*_*t*_	Binary GLM	410.46	1.02

6	*β*_0 _+ *β*_1_*Y*_*t*-2 _+ *β*_2_*pmax*_*t*_	Poisson GLM	407.40	0.97

Examination of the residual autocorrelation plots indicated that the working residuals autocorrelation plots were nearly identical, and all of them did not show any substantial remaining autocorrelation. Based on this, these three models appear to provide an adequate fit for the GDV occurrence data *Y*_*t*_.

The coefficients in these models were interpreted as either log odds (OR) in binary GLM models or multiplicative factors (MF) in Poisson GLM models. Table 2 contains the values of odds ratios and multiplicative factors for the lagged response *Y*_*t*-2 _and the external covariate for all the models from the Table 1. It also contains log-likelihood p-values for all of the odds ratios. In the names of the columns of Table 2, OR stand for the odds ratio and the MF for the multiplicative factor; the "other covariate OR" is either minimum daily atmospheric pressure on GDV day *pmin*_*t*_, the maximum daily atmospheric pressure on the GDV day *pmax*_*t *_or the maximum daily atmospheric pressure on the day before the GDV day *pmax*_*t*-1_. LL stands for the log-likelihood. Since the probability of GDV event on any given day is small and the values of the dog census are in the hundreds, the model can be fit as either binary GLM or Poisson GLM. Both approaches are employed for comparison purposes. There was very little difference between the binary GLM and Poisson GLM in the case for all the sets of covariates considered.

**Table 2:** Model Selection

Model	OR of *Y*_*t*-2_	LL p-value	Other Covariate OR/MF	LL p-value
1	3.1606	0.0595	1.0455	0.0891

2	3.0862	0.0640	1.0464	0.0833

3	2.9492	0.0741	1.0663	0.0064

4	2.8711	0.0807	1.0664	0.0062

5	3.0198	0.0693	1.0456	0.0633

6	2.9498	0.0741	1.0460	0.0606

Among the final group of models shown there were none that considered external covariates at the lag of more than 1 day. Such models were considered during the model selection stage, however. In all of those cases, the log-likelihood ratio p-value of the external covariate lags of more than 1 day was significantly above 0.10 whereas the coefficient of the 2-day lag of the response *Y*_*t*-2 _had the log-likelihood ratio p-value consistently below 0.10. Thus, the choice of covariates is not biased in favor of the lags of response *Y*_*t *_at the expense of the external covariate lags.

Based on these models, it seems that the factors that influence the rate of GDV most noticeably are thus the minimum daily atmospheric pressure on the day of GDV event, the maximum daily atmospheric pressure on the day of GDV event and the maximum daily atmospheric pressure on the day before the GDV event. The odds ratios/multiplicative factors of the external covariate in all models are slightly greater than 1 which indicates positive association between these factors and the probability of the GDV event on a given day. For example, the minimum daily atmospheric pressure on the day of GDV event has an odds ratio of 1.0455 for the binary GLM model. This suggests that for each increase of the minimum daily atmospheric pressure by 1 unit, the odds of the GDV case occurring on that day increase by the factor of 1.0455. For the respective Poisson model, the multiplicative impact factor is 1.0464 which means that for each increase of the minimum daily atmospheric pressure by 1 unit the probability of the day being a GDV day gets multiplied by the factor 1.0464. Note that all of the results for the external covariate should be interpreted conditionally on what happened two days before the observation day. Thus, it is more precise to say that the odds of GDV occurrence on any given day increase by the factor of 1.0617 for each unit increase in the minimum daily atmospheric pressure *given that we know whether there was or was not a case of GDV two days earlier*. The above statement cannot be made without knowing what happened two days before the day of observation. This is the important difference between our approach and that of the regular GLM modelling, whether binary GLM (logistic regression) or a Poisson model.

## Discussion and Conclusion

First, each of the models selected provided important information about the possible etiological factor of GDV and thus plays a useful role. It is not necessary to make a choice between them if the only purpose is to look into possible explanatory covariates for GDV occurrence. However, this becomes necessary if the forecasting of future GDV incidences is the main focus. We do not investigate this subject in the current paper.

Second, the variables identified as factors significantly influencing the rate of GDV occurrence were the minimum daily pressure on the day of GDV, the maximum daily pressure on the day of GDV and the maximum daily pressure on the day before the GDV event. Minimum daily pressure on the day of GDV was also identified as important and statistically significant factors in [7]. It is known that there is association between changes in barometric pressure and the onset of labor in humans and SIDS (sudden infant death syndrome) [11,12]. Less is known about the association between the atmospheric pressure and canine diseases. The current study suggests that the atmospheric pressure and changes in it may be the most important factors explaining the onset of GDV in dogs.

The value of a 2-day lag response in modeling for GDV was somewhat unexpected. This 2-day period may not necessarily correlate to a 48-hour period between events, however. The exact onset of the cascade of events leading to GDV is difficult to ascertain, and pathophysiological events leading to clinical signs may occur at different speeds in different dogs. Gastric distension has been noted to occur quickly in some dogs, and more slowly in others. The role of covariates that might be linked to delays in clinical manifestation, as well as the possible relationship(s) among meteorological phenomena during such a window of time, remain to be elucidated.

The approach using the logistic model for binary time series appears to be adequate in the case where there are at most a few daily observations with more than 1 GDV case. However, this may not be the case if a larger group of dogs is observed and, as a consequence, the number of days with more than one case of GDV becomes sizable. If this happens, Poisson GLM approach may have to be used exclusively.

A related paper [8] uses the latent variable modeling based on the hierarchical Bayes approach to incorporate the dependence between the observations. This approach is somewhat less flexible than the approach advocated in this paper. The reason is the fact that the hierarchical Bayesian approach requires certain specific prior assumptions, such as normality (or other specific distribution) of the data, that are not always easy to justify in practice. Also, their choice of the order 1 autoregressive process for the latent variable seems to be subjective and not based on any particular model selection algorithm whereas this research offers some insight into the possible selection mechanism based on criteria such as AIC. Finally, it is important to notice here that the time series approach is very natural when there are series of observations recorded over time. If it happens, such observations are almost always correlated; ignoring this correlation may result in the distorted inference concerning parameters of interest. In particular, it often results in exaggerated levels of significance for explanatory variables. Therefore, the time series based approach is, probably, the appropriate research tool in many clinical studies where the observations have been recorded over a period of time.

## Methods

### Data

The GDV occurrence data set consists of all recorded cases of GDV among the military working dogs (MWD) at the Lackland Air Force Base (LAFB) from January 1993 through December 1998. In each case, the breed of affected dog, its sex, date of birth, age at the onset of the disease and weight were recorded. All dogs were of one of three breeds: German Shepherd, Belgian Malinois, or Dutch Shepherd. The first recorded case of GDV occurred on Jan 6, 1993 and the last one on Dec 25, 1998. The total number of recorded cases (i.e. the days on which GDV case was registered) is 60. Out of 60, only two days involved more than one case of GDV; on both of them, there were 2 affected dogs. All kenneled dogs were checked by staff every 3 hours per organizational standard procedures. Cases were dogs that demonstrated abdominal swelling, tympani of the stomach, and radiographic evidence of gastric dilatation as determined by a veterinarian. Surgical intervention was initiated on all cases, either due to life-threatening condition or for non-emergency prophylactic gastropexy procedure.

The number of dogs under observation at LAFB was not constant but rather changing from month to month. The monthly dog census data was available Oct 1993 through Dec 1997 only, starting with 357 dogs in October 1993 and ending with 281 dog in December 1997. Because of unavailability of census data for 1998, GDV occurrence data for that year were not used.

A large database of weather data was assembled from the National Climatic Data Center at the Kelly Air Force Base, located immediately adjacent to LAFB. It contained hourly data on the wind direction, speed and wind gust; hourly temperature in Fahrenheit degrees, both adjusted and unadjusted for humidity and, finally, atmospheric pressure in inches of mercury, both adjusted to the sea-level, and unadjusted one (in millibars).

### Modeling approach

A number of possible models for the GDV occurrence in the dog population were considered. In all of them, the occurrence of GDV on a given day was coded using 1 for a GDV day or 0 for a non-GDV day and used as a response variable. Other quantities, such as, for example, atmospheric pressure and air temperature, were used as predictor variables. The binary response data lends itself to a number of possible approaches including binary GLM with the logistic link function (logistic regression) and a Poisson GLM with a log link function (Poisson regression).

Both response and covariates were recorded over time; this makes it reasonable to view both the response and covariates as time series. Therefore, instead of the usual generalized linear model that assumes the data are iid, we utilized the modified form of it where both response and covariates are autocorrelated over time.

Additionally, a time series approach is useful in this setting because it is quite likely that a number of weather-related (and possibly other) covariates are not accounted for; a large number of possible etiological factors makes it rather difficult to include all of them in one model. Besides air temperature and atmospheric pressure that were considered earlier, air humidity (either absolute or relative) may be one of the possible etiological factors. Humidity is recorded over time and usually exhibits noticeable autocorrelation. Typically, it is thought of as a time series; if the response variable is truly dependent on humidity, its omission causes the response variable to exhibit temporal autocorrelation. This line of thinking suggests that a time series model in the GLM framework may be better at describing GDV occurrence than the regular logistic GLM model considered in [7]. Other covariates that are commonly cited as the likely possible etiological factors of GDV, such as atmospheric pressure, air temperature and others, are also random time-dependent covariates (time series) themselves. Thus, the omission of *any one of them *is likely to induce additional autocorrelation in the response.

Out of several possible modeling approaches, this research used the one that is based on putting integer valued time series in the generalized linear model framework [13]. Unlike the earlier Markov chain approach, it does not cause the number of parameters to be estimated to grow exponentially with the sample size; it is also broad enough to encompass most of the practically important models. Note that neither Markov property nor stationarity have to be assumed when employing this approach. This is important since both of these properties may be difficult to verify in practice. The resulting models can be estimated using the same method (iterative reweighted least squares, IWLS for short) as regular generalized linear models; the only difference is that the results have to be interpreted conditionally on the past.

GDV occurrence on day *t *was denoted as *Y*_*t*_. Thus, *Y*_*t *_was binary. The covariates may include past and present values of explanatory variables as well as past values of *Y*_*t*_. The vector of all covariates was denoted as  where *p *is the number of covariates, *k *the number of explanatory variables lags and *q *the number of the past lags of the *Y*_*t *_considered; for each 1 ≤ *i *≤ *p *and 1 ≤ *j *≤ *k*,  represented the value of *i*th covariate at time *t *- *j*. The probability of a GDV case on any given day was defined as *p*_*t *_which is also a function of the covariate vector *z*_*t*_. There are at least two possible ways of modeling probability of GDV on a given day. The first choice is to use the binary GLM with the logit link function – effectively, a logistic regression model. The random component of the model then is a vector of binary values of *Y*_*t *_that are autocorrelated over time. The systematic component of the resulting logistic model becomes

(1)

where *α *is the intercept, *β *is the vector of coefficients and *p*_*t *_is the probability of the GDV event on a given day *t *that depends on the set of covariates **z**_*t*_. For each GDV day, the probability of the event is defined as the number of cases observed on that day divided by the total population of dogs recorded on that day. Because all of GDV probabilities were small in the study, it is also possible to model the probability of the GDV event on a given day *t *using the Poisson regression. This implies that the random component of the model is a vector of *Y*_*t *_that are viewed now as Poisson counts. The systematic component of the model relates the average number of cases on day *t μ*_*t *_to the covariates using the log link as

(2)

where, yet again, *α *is the intercept, *β *is the vector of coefficients and *μ*_*t *_depends on the set of covariates **z**_*t *_as before.

As a first step in the model selection procedure, the daily characteristics, such as max, min and mean, of the atmospheric pressure and air temperature were considered as possible covariates. The temperature was not adjusted for humidity. The lagged values of atmospheric pressure and/or temperature can be viewed as possible GDV etiological factors and thus additional explanatory variables as well. The likelihood-ratio tests were used to verify statistical significance of the model covariates. Explanatory variables that were to be used as a part of **z**_*t *_were chosen. Let us denote the minimum daily atmospheric pressure on a given day *pmin*_*t*_, maximum daily atmospheric pressure *pmax*_*t*_, maximum daily atmospheric temperature *tmax*_*t*_, and minimum daily atmospheric temperature *tmin*_*t*_. Past values (a day before) of the above were *pmin*_*t*-1_, *pmax*_*t*-1_, *tmax*_*t*-1 _and *tmint*_*t*-1_. The maximum hourly rise/drop in the atmospheric pressure on the day of GDV event was denoted *rp*_*t *_and *dp*_*t*_, respectively while the maximum hourly rise/drop in the temperature on the day of GDV event was denoted *rt*_*t *_and *dt*_*t*_. The dogs' breed was not considered since the dog population was rather homogeneous consisting of three large breeds routinely used as MWD. All non-event days were considered in this analysis as well.

The models were constructed using the process of stepwise forward selection. The covariates were added successively and the log-likelihood ratio for each new covariate is calculated. To avoid premature stop, even if the covariate did not add much to the descriptive ability of the model, as measured by its log-likelihood ratio statistic, the process continued until all of the covariates described earlier had been tried. The number of lags of *Y*_*t *_included in the models considered in this research was limited to 3 in order to guarantee parsimoniousness of the models. The residuals of each model can later be analyzed for autocorrelation patterns and additional lags added, if necessary. The coefficients of *Y*_*t*-1 _and *Y*_*t*-3 _had very large log-likelihood ratio p-values regardless of which external covariates were included in the model; more specifically, their log-likelihood ratio p-values exceed 0.1 everywhere and thus were excluded from the final models.

The models selected as final are shown in the Table (1). Except the minimum atmospheric pressure on GDV day and the maximum atmospheric pressure on the day of GDV event and on the day before the GDV event, all of the other external covariates display log-likelihood ratio p-values above the chosen threshold level of 0.10 are not included.

All of the models contained in the Table (1) are fit using the iterative reweighted least squares algorithm commonly applied to fitting of generalized linear models. Given that we are using the canonical link function log for the binary data, the iterative reweighted least squares algorithm coincides with the regular Newton-Raphson algorithm in this case.

## Authors' contributions

ML assisted in the study design, conducted the statistical modeling, and drafted the manuscript. GM conceived the study, acquired the data, assisted in the study design, and drafted the manuscript. Both authors have read and approved the final manuscript.
